# Active gaming in Dutch adolescents: a descriptive study

**DOI:** 10.1186/1479-5868-9-118

**Published:** 2012-10-02

**Authors:** Monique Simons, Claire Bernaards, Jantine Slinger

**Affiliations:** 1 Body@Work, Research Center Physical Activity, Work and Health, TNO- VU/VUmc, VU University Medical Center, Amsterdam, Netherlands; 2 Department of Health Sciences and the EMGO Institute for Health and Care Research, Faculty of Earth and Life Sciences, VU University, Amsterdam, Netherlands; 3 TNO, Expertise Center Life Style, Leiden, Netherlands

**Keywords:** Active video games, Adolescents, Physical activity behaviour, Physical activity guideline, Sedentary behaviour

## Abstract

**Background:**

Adequate levels of physical activity are part of a healthy lifestyle and in this way linked to better health outcomes. For children and adolescents, the physical activity guideline recommends at least 60 minutes of moderate-to-vigorous physical activity every day. However, many adolescents are not physically active enough and they spend a lot of their time on sedentary activities (such as video games). A new generation of video games that require body movements to play them, so-called "active games", could serve to increase physical activity in adolescents. The activity level while playing these games is comparable to light-to-moderate intensity physical activity. The current study aims to increase our understanding of 1) the demographic characteristics of adolescents who play active games regularly (≥ 1 hour per week) and non-regularly (<; 1 hour per week), 2) time spent on active games, 3) the contribution of active games to daily physical activity and 4) the type and amount of activities being replaced by active gaming.

**Methods:**

A cross-sectional survey was conducted in a Dutch internet panel, questioning adolescents in conjunction with one of their parents. A random sample of 320 households (with stratification on gender of the parent and the adolescent, the age of the adolescent and the region of the household) was selected that owned a console or application for active video games and that had a child aged 12 through 16 years. 201 child–parent couples (63% response) completed an internet survey with questions about demographics, physical activity and sedentary behaviour, and gaming behaviour. The questionnaire also contained questions designed to assess whether and how active gaming replaces other activities. Besides descriptive analyses, independent t-test, Pearson’s chi-square and Mann–Whitney test (when data were not normally distributed) were used for comparisons between regular and non-regular active gamers.

**Results:**

Eleven percent of the adolescents with an active game in their household never used the game. There were no significant differences in gender, education level (of adolescent and parent), ethnicity and sedentary behaviour between regular (n = 65) and non-regular active gamers (n = 114). Adolescents’ (regular and non-regular active gamers) meantime spent on active gaming was 80 (± 136) minutes a week; this potentially amounts to 11% of total physical activity. When time spent on active gaming was included in the calculation of the percentage of adolescents that met the physical activity guideline, the percentage increased significantly (p <; 0.05) from 67 to 73%. According to the adolescents, active gaming mainly replaces sedentary screen time such as TV viewing, internet and non-active gaming. Parental opinions concurred with this appraisal.

**Conclusions:**

The results of this study confirm the idea that active gaming may contribute to an active lifestyle in adolescents, primarily because it potentially contributes substantially to time spent on physical activity. Secondly, active gamers indicate that they spent time on active games which they would have spent otherwise on less active activities.

## Background

In all age groups, adequate levels of physical activity are part of a healthy lifestyle and linked to better health outcomes [1,2]. The physical activity guideline for Dutch children and adolescents recommends at least 60 minutes of moderate-to-vigorous physical activity a day [3-5]. Three quarters of the children and adolescents in the Netherlands do not meet this guideline [6]. The most noticeable decrease in physical activity occurs during adolescence [7,8].

Adolescents spend a lot of their time on sedentary activities and, in particular, video games [9-11]. In the Netherlands, 95% of adolescent boys and 81% of adolescent girls play video games; boys an average of 9.8 hours a week and girls 3.9 hours a week [11]. The amount of time spent on sedentary activities is linked to poorer health, regardless of the amount of time that someone is physically active [12,13]. In addition to the physical activity guideline, then, there is also a guideline for sedentary activities recommending that no more than two hours a day should be spent on recreational sedentary screen activities [14]. Both increasing physical activity and reducing sedentary behaviour are key public health targets [15,16].

A new generation of video games require body movements: "active games". In active video games, the gross body movement component replaces the hand control of traditional video games that requires only small finger and wrist movements. The player has to imitate the movements associated with, for example, boxing, tennis or dancing to play the game. Examples of active games are Microsoft Kinect, PlayStation®2 EyeToy^TM^ and Move (Sony), Dance Dance Revolution (Konami) and the Nintendo Wii^TM^ (Nintendo). Studies have shown that energy expenditure (EE) while playing active video games is substantially higher (100% through 400%) than EE during sedentary activities and is comparable to light to moderate physical activity (2 through 6 METs) [17-20]. Because adolescents are highly interested in computer games and more and more active computer games are becoming available, these games might contribute to an active and healthy lifestyle and they have attracted increasing interest from health-care professionals [21,22].

Active gaming can only have a potential effect on physical activity and health if adolescents are willing to spend time playing them. Currently, little is known about how much time adolescents spend on active gaming in a natural setting and about the demographic characteristics of adolescents who play active games. Baranowski et al. conducted a naturalistic study in younger children (9–12 years old) and showed that when children received two active (Wii) games they played it for 28 minutes per day in the first week and 8 minutes a day in week 12 [23]. Furthermore, we need to know more about the behavioural aspects of active gaming in a natural setting. In particular, how often and how long do adolescents play active games and what activities do they replace? If active games replace sedentary behaviour (such as traditional video games (non-active games)), active gaming will lead to an increase in the time spent on physical activity and reduce sedentary time (if other activities remain unchanged). In this way active gaming can result in an increase in the number of adolescents who meet the guidelines for physical activity and sedentary behaviour. However, it has not been demonstrated yet whether active gaming will replace less active forms of activity or whether it takes adolescents away from physical activities in which they already participate. The study of Baranowski et al. [23] among younger children did provide an indication that simply providing an active game to children does not result in a higher amount of physical activity. In this study the children were randomly assigned to either the active game condition (receiving two active Wii games) or the non-active game condition (receiving two non-active Wii games). The results showed no difference in physical activity between the two groups, indicating that simply acquiring active games does not spontaneously lead to an increase in physical activity in 9–12 years old children. No such study has been conducted yet among adolescents.

To develop interventions for reducing sedentary behaviour by replacing non-active behaviour with active gaming, it is important to fill these gaps in our knowledge about active gaming. This study therefore addressed the following questions: (1) what are the demographic characteristics of adolescents who play active games regularly (≥ 1 hour a week) and non-regularly (<; 1 hour a week)? (2) What is the contribution of playing active games to daily physical activity behaviour in adolescents who play active games occasionally? and (3) Does active gaming replace activities that are either more active (negative outcome) or less active (positive outcome) than active gaming in adolescents?

## Methods

The study population consisted of adolescents (12 to 16 years of age) with an active console in the household who play active games at home. To answer the questions addressed by this study, we questioned adolescents with one of their parents. The source population of the current study consisted of approximately 140,000 households from a Dutch internet panel. A representative sample of 4,817 households with at least one child in the 12–16 age category was first screened on the possession of a *console or application foractive video games (e.g.Nintendo Wii).* An active video game was defined as a game requiring physical activity involving more than finger and wrist movements to play. This selection yielded 2,937 households (60%) with an active console. Next, a random sample of 320 households was approached to complete the internet questionnaire. This sample was selected randomly, with stratification on gender of the parents and the adolescents, the age of the adolescents and the location of the household. Both adolescents and one of their parents were asked to answer a questionnaire in October 2010. Participants received a small incentive for filling in the questionnaire; the adolescents received 1.85 euro and the parents 1 euro. Valid responses were obtained from 201 child–parent pairs (a response rate of 63%).

### Measurements

#### Adolescents

The questionnaire for the adolescents contained questions about demographics (age, gender, ethnicity, and education level), physical activity behaviour, sedentary behaviour, active and non-active gaming behaviour. They were also asked which game consoles they owned and how long they had owned a console for active games. In addition, questions were asked to assess the replacement of other activities by active gaming.

### Physical activity and sedentary behaviour

Physical activity and sedentary behaviour were assessed with the Dutch Standard Physical Activity Questionnaire for youth (Indicators Monitor Youth Health) [24,25]. This questionnaire is commonly used in the Netherlands for assessing physical activity and sedentary behaviour in youth. The physical activity questions have recently been validated [26]. The sedentary activity questions have not been validated. However, they were developed by a panel of experts assuring face validity.

The physical activity questionnaire assesses the frequency and duration of four specific types of physical activity, namely (1) walking or cycling to school, (2) sports at school, (3) sports at a club, and (4) sports outside a club. Multiple-choice questions were used to establish frequency and duration per day in all four categories. Frequency was categorised as the number of days a week, ‘never’ or ‘I did not engage in that activity last week but I normally do’. Duration was assessed by numbers of minutes in different ranges that were averaged to calculate the average number of minutes spent on the specific physical activity. Duration in the category ‘walking and cycling to school’ was recorded as follows: less than 10 minutes a day = 5 minutes a day (on average), 10 to 20 minutes a day = 15 minutes a day, 20 to 30 minutes a day = 25 minutes a day, 30 to 60 minutes a day = 45 minutes, more than 60 minutes a day = 75 minutes a day. There was no question about walking or cycling for purposes other than commuting and so the duration of walking and cycling to school was then multiplied by 7/5 to extrapolate walking and cycling to school to walking and cycling for other purposes. This was based on the assumption that children who do not walk or cycle to school on schooldays will also not walk or cycle in the weekend [27]. For the categories ‘sports at a club’ and ‘sports outside a club’, duration was recorded as follows: less than 30 minutes a day = 15 minutes a day, 30 minutes to one hour a day = 45 minutes a day, one to two hours a day = 90 minutes a day, two to three hours a day = 150 minutes, more than three hours a day = 210 minutes a day. The duration for ‘sports at school’ was preset at 45 minutes. To calculate total physical activity time in minutes a week, the number of days reported for each category was multiplied by the average number of minutes a day. The answer categories for frequency - ‘never’ and ‘I did not engage in that activity last week but I normally do’ - were set at zero. Finally, the time spent in all four categories was totalled to calculate total time spent in physical activity. To meet the physical activity guideline for youth, adolescents need to be physically active at least 420 minutes a week (60 minutes a day). To determine whether adolescents met this physical activity guideline, it was assumed that the reported activities were performed at a moderate-to-vigorous intensity.

The questionnaire included two categories for recreational sedentary behaviour: 1) watching TV and DVD and 2) computer activities (internet, homework, games). As with the physical activity questions for both categories, multiple-choice questions were used to determine frequency and duration per day. The answer categories for frequency were the same as for the physical activity questions. Duration was determined in the same way as for the physical activity categories ‘sports at club’ and ‘sports outside a club’. To calculate total sedentary time in minutes per week, the same calculation was used as to calculate the time being physically active. To meet the sedentary behaviour guideline, adolescents should not spend more than 120 minutes a day on sedentary screen activities for entertainment purposes (computer, television) [14]. The cut-off for compliance with this guideline was therefore set at 840 minutes a week (120 minutes a day) on sedentary activities.

### Non-active and active game behaviour

The questions for assessing game behaviour were drafted for the current study and followed the same structure as the Dutch Standard Physical Activity Questionnaire. Frequency and duration per day of both active and non-active gaming were established. The answer categories for the questions regarding frequency were the number of days a week, ‘less than once a week’ and ‘I did not engage in that activity last week but I normally do’. Duration was recorded in the same way as the physical activity categories ‘sport at club’ and ‘sport outside a club’. To calculate the number of minutes a week spent on gaming, the same calculation was used as to calculate the time being physically active. So in adolescents who indicated that they did not play active/ non-active games in the last week but that they normally did or that they play active/ non-active games less than once a week, the calculation of the number of minutes per week spent on gaming resulted in zero.

### Replacement of activities by active gaming

To determine which activities are being replaced by active gaming, adolescents answered the following two multiple-choice questions: 1) “If you can remember when you didn't have an active game, what did you do with the time you now spend on active gaming?” and 2) “Imagine that you no longer have an active game: what will you do with the time you now normally spend on active gaming?” The answer options were: watching TV, Internet (chatting, MSN), non-active gaming, sports, homework, meeting friends, playing outside, making or listening to music, reading, sleeping. Adolescents could also answer that they have not played an active game in the last year or that they never play video games. Finally, adolescents were asked whether they thought this activity was more or less physically active than active gaming. These questions were specially developed for this study and have not been validated. The questions were pretested and this showed that adolescents and parents understood the questions and were able to answer them.

#### Parents

The questionnaire for the parents contained questions about demographics (age, relationship with adolescent (mother/father/carer), ethnicity and education level). Parents answered two questions about the replacement of the adolescent’s activities by active gaming: 1) “If you can remember when there was no active game in your household, what did your child do with the time he/she now spends on active gaming?” and 2) “Imagine that there will no longer be active games in your household: what do you expect your child would do with the time he/she now normally spends on active gaming?”. The answer options were the same as in the questions for adolescents, and the same question about the intensity of the replaced activity by comparison with active gaming was asked.

### Data preparation and analysis

Descriptive analyses were conducted for the adolescents who said they played active games occasionally (i.e. active gamers), so the non-users were excluded from the analyses. These adolescents were split up into two groups: 1) adolescents who played active games at least 1 hour a week, referred to as regular active gamers and 2) adolescents who played active games less than one hour a week, referred to as non-regular active gamers. The cut–off value of 1 hour a week of active gaming was based on the fact that individuals gain an average of 0.5 kg of excess body weight a year, which results from a positive energy balance of 70 kcal a week [28]. Studies showed that playing an active game resulted in an increase in energy expenditure of 60 kcal for one hour of playing Wii Sports [29] and 80 kcal for one hour of playing DDR compared to non-active gaming [30]. Replacement of a non-active game by an active gaming for one hour a week, would lead to an additional energy use of 70 kcal a week. Consequently, it is possible that playing active games for 1 hour a week may prevent excess weight gain.

Variables were tested for normality and non-normally distributed variables were log transformed. Independent sample t-tests and Pearson’s chi-square tests were used to compare regular and non-regular active gamers and to test gender difference in time spent on active and non-active gaming. If the log transformation did not lead to a normal distribution, the non-parametric Mann–Whitney test was performed to compare regular and non-regular active gamers. SPSS version 14.0 was used for the analysis and two-sided p-values <; 0.05 were considered significant.

The children and parents who participated in the study were registered at a Dutch Internet panel. All the children in this Internet panel provided consent to be in the internet panel and also their parents provided consent for their son or daughter and for their selves.

## Results

### Participants

Valid responses were obtained from 201 adolescent-parent couples (a response rate of 63%) with an active game in their household. Of these 201 adolescents, 22 (11%) never played active games. These 22 adolescents were excluded from analysis, which resulted in a total sample of 179 active gaming adolescents (active gamers), with one of their parents. Descriptive analyses were conducted for this group.

In Table 1, the demographic characteristics of regular active gamers (playing active games for one hour or more a week) are compared with those of non-regular active gamers (playing active games for less than one hour a week). The mean age was significantly (p = 0.004) lower in the regular active gamers group.

**Table 1:** **Demographic characteristics of regularand non-regular active gamers**

**Characteristic**	**All active gamers (n=179)**	**Regular active gamers (n=65)**	**Non-regular active gamers (n=114)**	**p-value**
Sex				0.601
-Boys (%)	50	52	48	
Mean age (years (SD))*	13.9 (1.3)	13.5 (1.3)	14.1 (1.3)	0.004
Education level				0.588
-% pre-vocational	42	46	40	
-% vocational	5	6	4	
-% advanced secondary	22	20	23	
-% pre-university	24	19	26	
-% other	8	9	7	
Parent education				0.871
-% low	17	15	18	
-% moderate	49	51	48	
-% high	34	34	33	
Ethnicity				0.172
% non-Dutch origin	7	11	5	

*Significant difference between regular and non-regular active gamers (p <; 0.05).

#### Sedentary behaviour and physical activity

The active gamers spent almost 4 hours a week playing non-active games, with boys spending significantly (p <; 0.001) more time (350 minutes a week) on these games than girls (100 minutes a week). Seventy-five per cent of the active gamers spent more than 120 minutes a day in sedentary behaviours and therefore did not meet the guideline for sedentary behaviour. There were no significant differences (p = 0.552) between regular and non-regular active gamers regarding meeting the guidelines for sedentary behaviour and physical activity.

The active gamers spent an average of 80 minutes a week playing active games. There was no significant difference (p = 0.120) in time spend playing active games between boys and girls. Eight per cent of the adolescents had not played active games in the past week but normally did, 42% played less frequently than once a week, 34% played once or twice a week and 10% three times a week, leaving 7% that played four to seven times a week. Of the adolescents who had played active games in the past week, 26% played for less than 30 minutes a day, 46% played for 30 to 60 minutes, 23% for 60 to 120 minutes and 5% for 120 to 180 minutes a day. Almost all participants (94%) owned a Nintendo Wii, 12% had a PlayStation EyeToy and 3% had a Dance Dance Revolution. Most adolescents (84%) said they had had an active game for one year or more.

Total time spend in physical activity behaviour, not taking into account active gaming, was on average 581 minutes a week. Based on these types of physical activity (not taking active gaming into account) 67% of the adolescents met the physical activity guideline. There were no significant differences (p = 0.496) in the percentages of regular and non-regular active gamers who complied with the physical activity guideline.

### Contribution of active games to daily physical activity

Table 2 shows that the adolescents spent an average of 660 minutes on total physical activity (including active gaming), with regular active gamers spending 798 minutes and non-regular active gamers 582 minutes a week on physical activity. When time spent on active gaming was included in the calculation of the percentage of adolescents who complied with the physical activity guideline, the percentage increased significantly (p <; 0.001) from 67% when active gaming was not taken into account to 73%. In the group of regular active gamers the percentage of adolescents that complied with the physical activity guideline was significantly (p = 0.009) higher compared to the percentage in the group of non-regular active gamers (85% versus 67%).

**Table 2:** **Time spent on categoriesof physical activity**

	**In all active gamers(N = 179)**	**Regular active gamers (N** **= 65)**	**Non-regular active gamers (N** **= 114)**	**p-values**
**Total physical activity includingactive gaming** (mean minutes a week (SD))	660 (354)	798 (384)*	582 (311)*	<;0.001
**-Transport** (mean minutes a week (SD))	242 (168)	211 (158)	259 (172)	0.072
**-Sport club** (mean minutes a week (SD))	154 (168)	164 (202)	148 (146)	0.833
**-Sport outside club** (mean minutes a week (SD))	105 (166)	132 (163)*	90 (167)*	0.006
**- Physical activity classesat school** (mean minutes a week (SD))	79 (36)	85 (37)	76 (35)	0.072
**-Active gaming** (mean minutes a week (SD))	80 (136)	205 (161)*	8 (17)*	<;0.001

*Significant difference between regular and non-regular active gamers (p <; 0.05).

An analysis of the relative contribution of each physical activity category to the total duration of physical activity showed that the largest contribution came from ‘transport’ (39%). Active gaming contributed 11% of total physical activity, which is close to the contribution from sport outside a club (such as playing soccer on the street) (13%) and physical activity classes at school (16%).

### Replacement of activities by active gaming

Tables 3 and 4 show which activities of adolescents were replaced by active games according to both the adolescents and one of their parents. Table 3 shows the activities in which adolescents engaged before there was an active game present in their household. Table 4 shows the activities adolescents would engage in if there was no longer an active game in their household.

**Table 3:** **Estimation of performed activitiesinstead of active gaming:adolescent perspective and parent’sperspective**

**Activity**	**According to adolescent (%)(n = 179)**	**According to the parent(%) (n = 174)**
TV viewing, internet or non-active gaming	71	77
Homework, listening to or making music, or reading	15	5
Sports or playing outside	5	13
I have not played active games in the past year/ My child has not played active games in the past year	1	1
Other	8	5

**Table 4:** **Activities expected to replaceactive gaming: adolescent perspectiveand parent’s perspective**

**Activity**	**According to adolescent (%)(n = 179)**	**According to the parent(%) (n = 174)**
TV viewing, internet or non-active gaming	71	78
Homework, listening to or making music, or reading	11	6
Sports or playing outside	4	7
I never play video games/ My child never plays video games or did not in the past year	2	1
Other	13	8

According to the adolescents, sedentary screen time such as TV viewing, internet and non-active gaming were the main activities that had actually been replaced, and that would be replaced, by active gaming. The parents agreed with the adolescents about this. About three-quarters of the adolescents (74-75%) said that the activity that was being replaced, or they thought they would be replaced, by active game playing was less intensive than active gaming. Most parents (73-78%) concurred.

## Discussion

The aim of the current study was to establish a clearer picture of the demographic characteristics of adolescents who play active games, to evaluate what impact access to an active game in the home environment has on the overall physical activity patterns of these active gamers, and to what extent active games can contribute to meeting the physical activity guideline. The target population of the current study consisted of adolescents with access to a console or application for active games in their home and who reported playing active games occasionally.

The results showed that the group of regular active gamers was slightly but significantly younger (i.e. 0.6 years) than the non-regular active gamers. Focus group studies showed that younger children like active games more than older children [31,32]. The 10–14 years old children in Dixon’s et al. study mentioned that playing active games would be less popular when they reached high school because it would not be seen as ‘cool’ [32]. This finding has implications for the age group active gaming interventions should target. Younger children might be a more suitable target group for active game interventions. It is important that game developers start devising active games that are attractive to older children.

No differences between regular and non-regular active gamers could be detected in other demographic factors. It should be pointed out that a sample of households was selected stratified by gender of the parents and the adolescents, the age of the adolescents and the locality of the households. The results for these demographic characteristics are therefore not representative for all households in the Netherlands with an active console or application.

The frequency of active gaming was not very high, with half of the adolescents playing active games less than once a week and one-third playing once or twice a week. On gaming days, a quarter of the adolescents played less than 30 minutes and almost half of the adolescents played for 30 to 60 minutes. The average total time spend on active gaming was 80 minutes a week, which is much less than the time spent on non-active gaming (224 minutes a week). The 80 minutes a week is comparable with the 71 through 109 minutes a week Maddison found for 10–14 year old children who received an active game upgrade [22]. Notably, these children were encouraged to meet the physical activity recommendations (60 min of moderate-to- vigorous physical activity on most days of the week) by supplementing periods of inactivity with active video game play and substituting periods of traditional non-active video game play with the active version. So the experimental situation in the study of Maddison et al. [22] is not totally comparable with the naturalistic setting of our study, where adolescents were not instructed or encouraged to use the active game. In the naturalistic study of Baranowski et al. it was found that 9–12 years old children spent 28 minutes per day in the first week and 8 minutes a day 12 weeks after receiving an active video game [23]. The data of our study show that simple access to an active game in the home environment is not enough to replace all time spent on non-active gaming by active gaming. The study of Baranowski et al. also provided an indication that simply acquiring a new active video game does not automatically lead to increased PA and a decrease in sedentary behaviour [23]. Interventions aiming at replacing non-active gaming by active gaming should involve more than just providing the active game. However, it is questionable whether it is realistic to expect the complete replacement of all non-active gaming. Even if only some sedentary time is replaced by active gaming, this may be a positive outcome with a potential impact on health. As stated earlier, an excess weight gain of 0.5 kg may be prevented by playing active games rather than sedentary activities at least 60 minutes a week.

In the current study, the relative contribution of time spent playing active games to the total amount of time spent on physical activity was 11%. This is comparable to the contribution of the time spent on playing sports outside a club. When the time spent on active gaming was included in the total amount of time that was spent on moderate to vigorous physical activity, the percentage increased significantly from 67 to 73%. It has to be noted that in order to contribute to compliance with the physical activity guideline, the intensity of physical activity should be moderate to vigorous. Research has shown that most active gaming is associated with moderate-intensity physical activity [20]. Intensity depends to a large extent on the game played, with higher intensity resulting from games that promote both upper and, in particular, lower body movement [17]. This means that not all active games have the potential to contribute to compliance with the moderate-to-vigorous physical activity guideline. It should also be noted that current evidence about the intensity of active gaming is all laboratory-based, and it is not known whether this intensity is matched during active gaming in the home environment. Single-session play in a laboratory may differ from play over time in a home environment, where active gaming is often episodic and unsupervised [17]. On the other hand, even active games that do not require moderate to vigorous intensity physical activity may still improve health by reducing sedentary behaviour or energy intake.

Since both adolescents and parents reported that active gaming replaced sedentary behaviour, it seems plausible to assume that active gaming can reduce sedentary time. In order to explore the possible effect of active gaming on the reduction of sedentary time, we subtracted active game play time from sedentary time. As a results, the percentage of adolescents that did not meet the sedentary behaviour norm (> 2 hours per day sedentary screen time) decreased significantly from 75% to 68% (data not presented). This exploration confirms the assumption that active games can still improve health even if they do not require moderate-intensity physical activity.

However the finding that active gaming replaces sedentary behaviour is only based on self perceived replacement behaviour. We did not find any differences in sedentary behaviour when we compared the groups of regular active gamers and non-regular active gamers with each other. It could also be that adolescents in our study had time for both sedentary activities and physical activity such as playing active games. A meta-analysis showed that sedentary behaviours and physical activity were only weakly associated, suggesting that adolescents have enough free time to be both very active and still have enough time to engage in high amounts of sedentary behaviour [33].

A recent active game intervention study did not show a significant treatment effect on time spent on moderate-to-vigorous physical activity measured by an accelerometer but did show a significant small effect on BMI and body composition in overweight and obese children [22].

A limitation of the current study is that it is a descriptive study and not an experimental study. The questions about the replacement of activities were not validated as it is difficult to validate these types of questions. The questions were specially developed for the current study and were pre-tested in adolescents and parents. Because the questions involved 1-year retrospective and future evaluations, participants might have found it difficult to provide an answer. But the pre-test showed that adolescents and parents understood the questions and were able to answer them. Conclusions based on these results should therefore be interpreted with care. On the other hand, the questions were asked both retrospectively and prospectively and they were put to both adolescents and parents. The results pointed in the same direction, strengthening the impression that active gaming mainly replaces inactive behaviour. Another limitation of this study is that physical activity was measured by questionnaires. Most questionnaires overestimate physical activity time because it is assumed that children are at least moderately active during the entire time they report being active. The questionnaire we used for calculating total time spent on physical activity is also known to overestimate physical activity [25]. Therefore, the percentage of 67% should not be interpreted as a indicator of how active our study population is compared to the total Dutch youth population. We also used a questionnaire similar to that of another study on 4–18 years old children [6]. According to that questionnaire, 96% of our sample did not meet the physical activity guideline. This percentage was higher than the 78% found in the study of Hildebrandt et al. [6] in general Dutch youth. This could indicate that our study population is very inactive compared to general Dutch youth. However, the two sets of data cannot be compared due to differences in age between the two samples (12–16 vs. 4–18 years old). It is known that during adolescence physical activity decreases, which could explain the higher percentage of not meeting the physical activity guideline found in our study. Objective measurements, such as accelerometers, could be used in future studies to better estimate physical activity levels as well as the sedentary time.

A strong element of the current study is that it is the first to establish a picture of the demographic characteristics of adolescents who play active games and of the behavioural aspects of active gaming. In particular, how often and how long adolescents play active games and what activities are replaced by active gaming. The current study provides a good indication of the impact of access to an active game at home on the overall physical activity of active gamers in an entirely natural setting, with no rules or restrictions resulting from an intervention programme.

Even so, more research is necessary to investigate the impact of active games in a home environment on sedentary behaviour and physical activity over time. Long-term randomised controlled trials will be needed to investigate whether there is a cause-effect relationship between active games and physical activity. Furthermore, it is important to experimentally test the impact of the introduction of active games on other leisure activities and to investigate whether playing active games replaces more vigorous or more sedentary activities. More studies such as the trial of Baranowksi [23] using objective measurements for physical activity should be conducted. Future studies should not only focus on the Wii but also on other active games such as Microsoft Kinect and PlayStation^©^Move and should also focus on other age groups (such as adolescents). Finally, besides accelerometers worn on the hip, additional measurements for physical activity should be included because accelerometers worn on the hip might not be sensitive enough to measure overall physical activity during active gaming as many active games require mainly upper arm movements [22]. For example additional accelerometers could be worn on the wrist or additional heart rate monitors could be used for measuring physical activity and should also be worn more frequently and continuously to make sure to capture overall physical activity.

## Conclusions

The results of the current study reinforce the idea that active gaming may potentially contribute to an active lifestyle in adolescents. Active gamers seem to spend a substantial amount of time on playing active games which they otherwise would have spent on less active pursuits.

## Consent

The children and parents who participated in the study were registered at a Dutch Internet panel. All the children in this Internet panel provided consent to be in the internet panel and also their parents provided consent for their son or daughter and for their selves.

## Competing interests

The authors declare that there are no competing interests.

## Authors’ contributions

JS obtained funding. MS and JS designed the study. MS performed statistical analyses. All authors participated in the interpretation of analyses. MS drafted the manuscript. JS and CB critically revised the manuscript. All authors read and approved the final manuscript.

## References

[B1] Bouchard C Shephard RJ Stephens T Physical activity, fitness, and health: International proceedings and consensus statement 1994 Human Kinetics Publishers, Champaign, Illinois

[B2] US Department of Health and Human Services 1996 10.3109/15360288.2015.1037530 26095483

[B3] Kemper HGC Ooijendijk WTM Stiggelbout M Consensus over de Nederlandse Norm voor Gezond Bewegen Tijdschr Soc Gezondheidsz 2000 78 180 183

[B4] Strong WB Malina RM Blimkie CJ Daniles SR Dishman RK Gutin B Hergenroeder AC Must A Nixon PA Pivarnik JM Rowland T Trost S Trudeau F Evidence based physical activity for school-age youth J Pediatr 2006 146 6 732 737 15973308 10.1016/j.jpeds.2005.01.055

[B5] US Department of Health and Human Services Physical Activity Guidelines for Americans 2008 http://www.health.gov/paguidelines/default.aspx

[B6] Hildebrandt VH Chorus AMJ Stubbe JH Trendrapport bewegen en gezondheid 2008/2009 [Trend report physical activity and health 2008/2009] 2010 Leiden, TNO

[B7] Van Mechelen W Twisk JW Post GB Snel J Kemper HC Physical activity of young people; the Amsterdam longitudinal growth and health study Med Sci Sports Exerc 2000 32 1610 1616 10994913 10.1097/00005768-200009000-00014

[B8] Riddoch CJ Anderse LB Wedderkoop N Harro M Klasson-Heggebo L Sardinha LB Physical activity levels and patters of 9- and 15- year old European children Med Sci Sports Exerc 2004 16 1 86 92 14707773 10.1249/01.MSS.0000106174.43932.92

[B9] Rideout VJ Foehr UG Roberts DF Generation M²: Media in the Lives of 8- to 18-Years Olds 2010 Kaiser Family Foundation, Menlo Park, CA

[B10] Fromme J Computer games as a part of children’s culture Int J Comput Game 2003 3 (1)

[B11] The Netherlands National Gamers Survey 2009 http://www.newzoo.com/press/TodaysGamers_SummaryReport_NL.pdf. Accessed December 6, 2010

[B12] Boone JE Gordon-Larsen P Adair LS Popkin BM Screen time and physical activity during adolescence: longitudinal effects on obesity in young adulthood Int J Behav Nutr Phys Act 2007 4 26 10.1186/1479-5868-4-26 17559668 PMC1906831

[B13] Hamilton MT Hamilton DG Zderic TW Role of low energy expenditure and sitting in obesity, metabolic syndrome, type 2 diabetes, and cardiovascular disease Diabetes 2007 56 11 2655 2667 10.2337/db07-0882 17827399

[B14] American Academy of Pediatrics Children, adolescents, and television: committee on public education Pediatrics 2001 107 423 426 11158483 10.1542/peds.107.2.423

[B15] DeMattia L Lemont L Meurer L Do interventions to limit sedentary behaviors change behavior and reduce childhood obesity? A critical review of the literature Obes Rev 2007 8 1 69 81 10.1111/j.1467-789X.2006.00259.x 17212797

[B16] Reilly JJ McDowell ZC Physical activity interventions in the prevention and treatment of paediatric obesity: systematic review and critical appraisal Proc Nutr Soc 2000 62 3 611 619 14692597 10.1079/PNS2003265

[B17] Biddiss E Irwin J Active video games to promote physical activity in children and youth: a systematic review Arch Pediatr Adolesc Med 2010 164 7 664 672 10.1001/archpediatrics.2010.104 20603468

[B18] Daley AJ Can exergaming contribute to improving physical activity levels and health outcomes in children? Pediatrics 2009 124 2 763 771 10.1542/peds.2008-2357 19596728

[B19] Simons M de Vries SI Jongert T Verheijden MW Energy expenditure of three public and three home based active video games in children Comput Entertain In press

[B20] Barnett A Cerin E Baranowski T Active video games for youth: a systematic review J Phys Act Health 2011 8 5 724 737 21734319 10.1123/jpah.8.5.724

[B21] Foley L Maddison R Use of active video games to increase physical activity in children: a (virtual) reality? Pediatr Exerc Sci 2010 22 1 7 20 20332536 10.1123/pes.22.1.7

[B22] Maddison R Foley L Ni Mhurchu C Jiang Y Jull A Prapavessis H Hohepa M Rodgers A Effects of active video games on body composition: a randomized controlled trial Am J Clin Nutr 2011 May 18. Epub ahead of print 10.3945/ajcn.110.009142 21562081

[B23] Baranowski T Abdelsamad D Baranowski J O'Connor TM Thompson D Barnett A Cerin E Chen TA Impact of an active video game on healthy children's physical activity Pediatrics 2012 129 3 e636 e642 10.1542/peds.2011-2050 22371457 PMC3289528

[B24] Indicators Monitor Youth Health. Standard Questionnaire for Physical Activity Indicatoren voor de Monitor Jeugdgezondheid. Standaardvraagstelling Bewegenwww.monitorgezondheid.nl/jeugdindicatoren.aspx. (in Dutch)

[B25] Singh AS Chin APMJ Kremers SP Visscher TL Brug J van Mechelen W Design of the Dutch Obesity Intervention in Teenagers (NRG-DOiT): systematic development, implementation and evaluation of a school-based intervention aimed at the prevention of excessive weight gain in adolescents BMC Publ Health 2006 6 304 10.1186/1471-2458-6-304 PMC1769372 17173701

[B26] Schokker DF Hekkert KD Kocken PL Van den Brink CL De Vries SI Measuring physical activity in children: questionnaires versus accelerometers. (Meten van lichamelijke activiteit van kinderen: vragenlijsten versus versnellingsmeter 2012 TSG (in Dutch)

[B27] Van Wieringen Point of view physical activity promotion by youth healthcare. (Standpunt Beweegstimulering door de Jeugdgezondheidszorg) 2009 RIVM in Dutch

[B28] NHS-NRG Prevention of weight gain and guidelines for weight control in the Netherlands. (Preventie van gewichtsstijging en richtlijnen voor gewichtsbeheersing in Nederland) 2007 (in Dutch)

[B29] Graves L Stratton G Ridgers ND Cable NT Comparison of energy expenditure in adolescents when playing new generation and sedentary computer games: cross sectional study BMJ 2007 335 7633 1282 1284 10.1136/bmj.39415.632951.80 18156227 PMC2151174

[B30] Lanningham-Foster L Jensen TB Foster RC Redmond AB Walker BA Heinz D Levine JA Energy expenditure of sedentary screen time compared with active screen time for children Pediatrics 2006 118 6 e1831 e1835 10.1542/peds.2006-1087 17142504

[B31] Simons M de Vet E Hoornstra S Brug J Seidell J Chinapaw M Adolescents’ views on active and non-active video games: a focus group study Games Health J 2012 1 3 211 218 10.1089/g4h.2011.0032 26193439

[B32] Dixon R Maddison R Ni Mhurchu C Jull A Meagher-Lundberg P Widdowson D Parents' and children's perceptions of active video games: a focus group study J Child Health Care 2010 14 2 189 199 10.1177/1367493509359173 20203134

[B33] Marshall SJ Biddle SJ Gorely T Cameron N Murdey I Relationships between media use, body fatness and physical activity in children and youth: a meta-analysis Int J Obes 2004 28 1238 1246 10.1038/sj.ijo.0802706 15314635

